# Transinfected *Wolbachia* have minimal effects on male reproductive success in *Aedes aegypti*

**DOI:** 10.1186/1756-3305-6-36

**Published:** 2013-02-11

**Authors:** Andrew P Turley, Myron P Zalucki, Scott L O’Neill, Elizabeth A McGraw

**Affiliations:** 1 The School of Biological Sciences, Monash University, Clayton, Vic, 3800, Australia; 2 The School of Biological Sciences, The University of Queensland, Brisbane, Qld, 4072, Australia; 3 The Institute for Molecular Biosciences, The University of Queensland, Brisbane, Qld, 4072, Australia

**Keywords:** Mosquito, Fecundity, Symbiont, Multiple mating, Larval nutrition *Wolbachia*

## Abstract

**Background:**

*Wolbachia* are maternally inherited endosymbiotic bacteria that manipulate the reproductive success of their insect hosts. Uninfected females that mate with *Wolbachia* infected males do not reproduce due to cytoplasmic incompatibility (CI). CI results in the increased frequency of *Wolbachia*-infected individuals in populations. Recently, two *Wolbachia* strains, the benign *w*Mel and virulent *w*MelPop have been artificially transinfected into the primary vector of dengue virus, the mosquito *Ae. aegypti* where they have formed stable infections. These *Wolbachia* infections are being developed for a biological control strategy against dengue virus transmission. While the effects of *Wolbachia* on female *Ae. aegypti* have been examined the effects on males are less well characterised. Here we ascertain and compare the effects of the two strains on male fitness in resource-limited environments that may better approximate the natural environment.

**Methods:**

A series of population mating trials were conducted to examine the effect of *Wolbachia* infection status (with strains *w*Mel and *w*MelPop) and male larval nutrition on insemination frequency, remating rates, the fecundity of females, the hatch rates of eggs and the wing length and fertility of males.

**Results:**

*w*Mel and *w*MelPop infections reduce the fecundity of infected females and *w*MelPop reduces the viability of eggs. Low nutrition diets for males in the larval phase affects the fecundity of *w*Mel-infected females. Neither strain of *Wolbachia* affected sperm quality or viability or the ability of males to successfully mate multiple females.

**Conclusions:**

The benign strain of *Wolbachia*, *w*Mel causes similar reductions in fecundity as the more virulent, *w*MelPop, and neither are too great that they should not still spread given the action of CI. The ability of *Wolbachia*-infected males to repeat mate as frequently as wildtype mosquitoes indicates that they will be very good agents of delivering CI in field release populations.

## Background

*Wolbachia* are maternally inherited endosymbiotic bacteria that naturally infect over half of all insect species [1]. Two different *Wolbachia* strains, native to *Drosophila melanogaster*, have been artificially transinfected into the primary vector of dengue virus, the mosquito *Aedes aegypti,* where they have formed stable infections [2,3]. These artificially created lines are the cornerstone of a biological control program to limit dengue transmission to humans that has progressed from the bench [2] to field cages [3] to open field releases [4]. In Far North Queensland, Australia, the site of the first release, the ability for *Wolbachia* infections to spread and be maintained long-term in wild populations has been demonstrated [4]. In the next few years, the ability of *Wolbachia* infected mosquitoes to reduce disease transmission in human populations will be tested in countries where dengue is endemic including locations in Vietnam, Indonesia and Brazil. In preparation for these releases, mathematical models are being employed to predict how *Wolbachia* will spread in populations of the vector and its consequences for dengue epidemiology. Key components of these models include the effects of *Wolbachia* on host fitness that can vary between strains. Understanding these differences are critical to ensure the correct strain is chosen for release sites that will maximise both spread and disease control outcomes.

The two *Wolbachia* strains, *w*Mel and *w*MelPop differ in their effects on hosts and how they may be utilised to limit disease transmission. Both strains induce Cytoplasmic Incompatibility (CI) [2,3]. This reproductive manipulation favours *Wolbachia* infected females over uninfected in wild populations hence *Wolbachia* infections tend to spread [5,6]. CI is what first raised interest in *Wolbachia* as a biocontrol agent – because it can effectively drive itself into populations [7]. Both *w*Mel and *w*MelPop also possess a characteristic that was only recently discovered, the ability to limit the replication of dengue virus inside the mosquito [3,8,9]. While the midgut of the mosquito almost always becomes infected following a dengue-laden blood meal, few mosquitoes progress to the point of secreting dengue in the saliva which is required for human transmission to occur. Interestingly, this same ability to block pathogen infection has been demonstrated for other viruses [8,10,11], the filarial nematode, *Brugia pahangi*[12] and an avian model of the malaria parasite, *Plasmodium gallinaceum*[8]. The *w*MelPop strain alone causes significant life shortening. Mosquitoes infected with the *w*MelPop strain exhibit on average a lifespan that is half that of uninfected mosquitoes [2]. The strain was initially selected for transinfection because of the life shortening it caused in its native *Drosophila melanogaster*[13]. Dengue, like many other pathogens is not immediately transmissible following consumption of an infected blood meal by a mosquito. There is a delay while the virus migrates to the salivary glands (7–10 days). The result is that only older mosquitoes surviving past this age can transmit virus, making lifespan reduction an attractive means for reducing transmission at the population level [7].

In addition to these traits, *Wolbachia* has been shown to also affect host fecundity, fertility, locomotion, dispersal, immunity, foraging and mating behaviours in a range of insects [14-19]. The frequency and magnitude of these changes can be specific to both host species and *Wolbachia* strains, in some cases enhancing fitness in one host but reducing it in another [19-21]. Studies so far in *Ae. aegypti* have shown that *w*MelPop infections can reduce a females’ fecundity, egg viability and ability to blood feed [2,22-24]. The *w*Mel strain is relatively benign causing only a 10% reduction in longevity [3]. Given *Wolbachia’s* maternal inheritance, the nature of standard fitness assays and the fact that only female mosquitoes transmit disease, it is perhaps not surprising that the majority of this work has been female focused. Males have a unique and key role to play in populations because they are the agents of CI, blocking the successful reproduction of uninfected females during mating. Offspring of infected females then by default form a greater proportion of the subsequent generation and *Wolbachia* spreads. So it is critical that *Wolbachia* infected males are sufficiently competitive relative to wild type males. While female *Ae. aegypti* most often only mate once (but see [25]), males are more likely to attempt to mate multiple times [26-28]. The ability of individual males to successfully mate multiple females could speed the efficacy of CI in populations.

A number of male-based effects of *Wolbachia* have been characterised in *Drosophila simulans*. Snook *et al.* showed that *Wolbachia-*infected males produced sperm cysts at a slower rate than uninfected males, which resulted in infected males producing approximately 40% fewer sperm cysts than uninfected males [29]. This effect became more extreme as the flies aged. In addition, non-virgin *Wolbachia*-infected *D. simulans* males sire fewer progeny due to production of less competitive sperm [17] leading to reduced egg viability [29]. These phenotypes could result in fewer females being fully inseminated and an increased proportion of females being partially inseminated by *Wolbachia-*infected males. In mosquitoes, male mating performance has recently been shown not to vary following artificial transinfection of *Wolbachia,* however, in this case the donor insect for the *Wolbachia* was a sister mosquito species [30]. In cases where *Wolbachia* has been transferred from a phylogenetically distant host species, however, more extreme effects might be expected in the novel host according to host:parasite theory [21,31,32].

Lastly, while standard measures of fitness are employed in the laboratory under ideal conditions it is often difficult to generalise their meaning to the field, where nutritional availability is likely to be lower. The nutrition of larvae is an important determinant of adult mosquito fitness. Mosquitoes that grow in low-nutrition larval environments develop more slowly and have smaller body sizes and teneral reserves as adults compared to those reared in high-nutrition environments. In most cases, mosquitoes that grow in low-nutrition larval environments have reduced performance compared to their larger counterparts, including: reduced dispersal, flight potential [33,34], blood-meal size, fecundity [35-38], male fertility [39], immunity [40-42], survival [33,43,44], host-seeking behaviours [43,45,46] and mating success [39,47-49]. Recently, Yeap *et al*. [50] showed that *Wolbachia* infection could interact with larval nutrition to modify larval development times and the wing size of *Ae. aegypti* males. However, it is unknown how this interaction might affect performance of infected mosquitoes in the field. An understanding of these interactions may be used to inform breeding practices of mosquitoes prior to field release as was the case for medflies being mass reared for sterile insect release [51]. In this program, lab-reared males exhibited poor competitiveness [52] but supplementing their diets with protein improved courting and copulation frequencies, increased insemination and fertilisation success and decreased the likelihood of females remating [51,53,54].

Here we investigate the effects of two strains of *Wolbachia – w*Mel and *w*MelPop – on the ability of males to successfully mate and reproduce with *Wolbachia* infected and *Wolbachia* free females. Trials with unequal (1:5) male-to-female ratios were conducted to test the potential of males to successfully mate with multiple females and to test the influence of this behaviour on the fecundity of females and their egg viability. Remating trials were conducted to test whether males could replenish seminal supplies and successfully mate with subsequent females. Lastly, males were reared on low-nutrition larval diets to test for potential interactions between nutritional status and *Wolbachia* infection. Response variables measured included the following traits: wing length, quantity and quality of sperm and the ability of males to successfully mate and reproduce with females.

## Methods

### Mosquito strains

The *Wolbachia-*infected mosquitoes employed in the development assays were from PGYP1, an inbred line of *Ae. aegypti* infected with the *w*MelPop strain of *Wolbachia* in 2008. Uninfected matched controls were of PGYP1.tet, a line of PGYP1 mosquitoes previously cured of *Wolbachia* through treatment with tetracycline [2]. Subsequently, outcrossed versions of these *Wolbachia* infected lines, A.PGYP1.out (*w*MelPop infected) and MGYP2.out (*w*Mel infected), were generated using a scheme of mating to wild-type males as developed by Yeap *et al*. [50]. Wing length, mating and reproduction experiments were conducted using the outcrossed lines. The wild-type (control) line consisted of *Ae. aegypti* reared from eggs collected from breeding sites in Cairns, Queensland, Australia (16°51^′^S, 145°45^′^E), in 2009–2011. Male remating experiments and male fertility assays tested mosquitoes from the A.PGYP1.out and wild-type lines of mosquitoes, as described above.

### Mosquito rearing and nutrition

All mosquitoes were reared in a climate-controlled insectary at 26 ± 1°C, RH 60 ± 5% with 12h:12h light/dark cycle. *Ae. aegypti* eggs were submerged in distilled water in a flask and connected to a vacuum for 30 minutes to induce hatching. Larvae for development assays were reared in 500ml of distilled water at a density of 25 larvae per tray. The control larval diet consisted of 2mg of TetraMin Tropical Fish Tablets per larvae per day. Low-nutrition diets consisted of 0.25mg, 0.2mg or 1.5mg of TetraMin Tropical Fish Tablets per larvae per day. The low nutrition treatment (0.25 mg food per day per larva) was based on a previous study showing increases in developmental time under this feeding regime [50].

Larvae for forewing length, mating and reproduction experiments and male fertility assays were reared in 3L of distilled water at a density of 150 per tray. For high-nutrition rearing, larvae were fed 2mg of TetraMin Tropical Fish Tablets per larva per day. For low-nutrition rearing, larvae were fed 0.2mg of TetraMin Tropical Fish Tablets per larva per day. Due to the delayed development of larvae reared on low nutrition, eggs for low-nutrition larvae were hatched two days before eggs for high-nutrition larvae. To obtain virgin mosquitoes, pupae were sorted by sex, based on size and shape, and separated. Adults were maintained in 30x30x30cm cages at a density of 450 mosquitoes per cage, with access to 10% sucrose solution.

### Development assays

The effect of *Wolbachia* infections and larval nutrition on the development time of *Ae. aegypti* larvae was assessed. Two replicate trays of PGYP1 and PGYP1.tet larvae were reared on each of four nutritional regimes (2mg, 0.25mg, 0.2mg and 0.15mg per larvae per day). Development time was estimated by counting the number of pupae, in each tray, for 30 consecutive days. Each day, all pupae were removed and remaining larvae fed an adjusted amount of food.

### Wing length

Wing lengths of 10 males from each of the MGYP1.out, PGYP1.out and wild-type lines, reared on high or low-nutrition (0.2mg/larva/day) larval diets were measured*.* At five days of age the right wings were removed from the mosquitoes and measured from the axillary incision to the wing tip, under a dissecting microscope using an eyepiece micrometer.

### Mating and reproduction experiments

The mating success of males was examined by investigating the effect of *Wolbachia* infection and male larval nutrition on the number of females successfully inseminated, the fecundity of females and the hatch rate of their eggs. Males were reared on a high (2mg/larva/day) or low-nutrition (0.2mg/larva/day) larval diet and adults were reared to five days of age. Females were reared on high-nutrition larval diets only and aged to five days old. To test the impact of the infection status of males, five lines (mating combinations) were separately tested: MGYP2.out females x MGYP2.out males (MOMO), MGYP2.out females x wild-type males (MOWT), A.PGYP1.out females x A.PGYP1.out male (POPO), A.PGYP1.out females x wild-type males (POWT) and wild-type males x wild-type females (WTWT). The incompatible cross between *Wolbachia-*infected males and uninfected females was not tested [2,3].

In each experiment, 10 five-day-old males and 50 five-day-old females were knocked down by chilling and transferred into a 645mm^3^ cage. Males and females cohabited for 24 hours before all mosquitoes were aspirated out of the cage. To facilitate the development of eggs, all females used in this experiment were offered a human forearm (APT) from which to blood feed the day before and during each experiment for 15 minutes. Females were transferred into individual 40ml tubes, containing water and filter paper, for oviposition. Females were allocated seven days in oviposition tubes before they were examined for the presence or absence of eggs. Females that laid fewer than 10 eggs were excluded from the results to avoid the possibility of autogenous egg batches [55]. All other eggs were counted to determine fecundity.

Hatch rates of eggs were determined by transferring paper and water from oviposition tubes into trays containing 250ml water and 30mg TetraMin Tropical Fish Tablets. Eggs were left for 48 hours to hatch before the larvae in each tray were counted. As some eggs may not have matured during the first hatch, egg papers were dried down and stored for three days before immersing a second time. The numbers of larvae from the first and second hatch were combined to determine the hatch rate of eggs.

In some egg batches all eggs failed to hatch. When this occurred, the females that laid the eggs were dissected to confirm the presence or absence of sperm in their spermathecae. Using fine forceps, spermathecae were removed from females under a dissecting microscope. To stain the sperm, spermathecae were placed into 4μl of 1:4 Propidium Iodide:H_2_0 (Invitrogen-Molecular Probes) solution and a cover slip placed on top of the slide, bursting the spermathecae. The presence or absence of sperm was determined using a fluorescence microscope (Zeiss Axio Imager, Carl Zeiss MicroImaging) equipped with a live/dead filter (Chroma Technology Corp.). The number of females successfully inseminated was calculated by adding the number of females that laid viable eggs and the number of inseminated females that laid eggs that did not hatch.

### Male remating experiments

Males from the previous mating and reproductive success experiments were exposed to a second cohort of females. Previous research suggested that male *Ae. aegypti* could replenish seminal stocks within three days after mating [56]. Wildtype and A.PGYP1.out males were maintained on 10% sucrose for four days after the first trials. Due to males dying during this renewal period, the remating experiments involved randomly selecting only five, of the possible 10, males from the first trials and transferring these mosquitoes into cages containing only 25 five-day-old females. For all males, the infection status of females in their remating experiment was the same as those in their first mating success experiment. The experimental procedure was as per the mating and reproductive success experiments above. Males and females again cohabited for 24 hours and females were offered blood meals. Females were then transferred to individual oviposition tubes and fecundity and hatch rates of eggs were recorded.

### Male fertility assays

This assay examined the quantity and viability of sperm of 10 male mosquitoes from the A.PGYP1.out and wild-type lines reared on high (2mg/larva/day) and low-nutrition (0.2mg/larva/day) larval diets. Sperm were stained using an Invitrogen Live/Dead Sperm Viability Kit (L-7011) (Invitrogen-Molecular Probes) using the following protocol. For each day of experiments, a 50-fold dilution of the SYBR 14 stock solution in HEPES buffer (10mM HEPES, 150mM NaCl, 10% BSA, pH 7.4) was prepared and a master dye-mix was then created by combining 1:1:3 SYBR 14 dilution, Propidium Iodide, and H_2_0. The master dye-mix was stored on ice and in the dark for the duration of the day’s dissections. To each sperm dilution, 5μl of master dye-mix was added and mixed gently but thoroughly with a pipette.

Previous research has shown that sperm quantity can be reliably estimated in *Ae. aegypti* by counting multiple aliquots of a sperm sample (10 of 40) [39]. Estimates of sperm quantity were, therefore, determined by spotting 10 5μl aliquots of sperm dilution onto a multiwell slide. Slides were left to air dry at room temperature and in the dark. Upon examination, 5μl 1x PBS was added to each well of the slide and a cover slip carefully placed onto the slide. Samples were examined using a fluorescence microscope (Zeiss Axio Imager, Carl Zeiss MicroImaging) equipped with a live/dead filter (CHROMA Technology Corp.). The total count of the 10 aliquots was averaged and multiplied by 40 to estimate the total quantity of sperm of each male.

Sperm viability protocol was as per sperm quantity assays with the following exceptions. The viability of sperm was determined by spotting 10 2μl aliquots of sperm dilution onto a multiwell slide and incubating at room temperature in the dark for 10 minutes. A cover slip was then placed over the slide and each well immediately examined using a fluorescence microscope (Zeiss Axio Imager, Carl Zeiss MicroImaging) equipped with a live/dead filter (Chroma Technology Corp.). The viability of sperm was calculated by dividing the total number of live sperm by the total (live + dead) number of sperm counted from each male.

### Statistical analysis

All data were checked for normality by distribution model fitting and variances checked for equality. The wing length, number of females that laid eggs, fecundity and sperm quantity and viability were normally distributed and were analysed with general linear models, with significant factors examined using appropriate *t*-tests with Holm-Bonferroni correction for multiple comparisons. Larval development times, hatch rates of eggs and remating success of males data were not normally distributed and could not be transformed; therefore these data were analysed using generalized linear models and significant factors were examined using Kolmogorov-Smirnov tests with Holm-Bonferroni correction. All data analysis was conducted using SPSS (IBM).

Approval for blood feeding by human volunteers for maintenance of the mosquito colony was granted by The University of Queensland Medical Research Ethics Committee (#200700137).

## Results

### Larval development

*Wolbachia* infections did not affect the development of larvae. A generalized linear model (Poisson with log link error) showed that nutrition (Wald = 79.425, *P* < 0.001), but not mosquito line (Wald = 0.562, *P* = 0.454) and interactions between these factors (Wald = 0.759, *P* = 0.859), affected larval development. Compared to control nutrition, all low-nutrition regimes significantly increased the median larval development time (0.25mg: Z = −11.61, *P* <0.001; 0.2mg: Z = −12.093, *P* <0.001; 0.15mg: Z = −12.469, *P* < 0.001).

### Wing length

There was a significant difference between the wing length of low and high-larval nutrition males (F = 409.439, *P* < 0.001), but there was no effect of *Wolbachia* infection (F = 0.537, *P* = 0.587) or interaction between the factors (F = 2.789, *P* = 0.070). Males reared on low nutrition had smaller wings ( $\bar{x}$ = 3.873, SEM = 0.029) than males reared on high nutrition ( $\bar{x}$ = 4.716, SEM = 0.030).

### Number of females successfully inseminated

Male larval nutrition, but not *Wolbachia* infections, influences the number of females that lay eggs. The number of females successfully inseminated was affected by the larval nutrition of the male (F = 10.143, df = 1, *P* < 0.05). Fewer females were inseminated by males reared on low larval nutrition diets ( $\bar{x}$*=* 3.9, SEM = 0.26) than by males reared on high nutrition diets ( $\bar{x}$ = 5, SEM = 0.24). Mosquito line (F = 2.48, df = 4, *P* = 0.065) and interactions between mosquito lines and male larval nutrition (F = 0.114, df = 4, *P* = 0.976) did not affect the number of females that laid eggs.

### Fecundity

**Figure 1:**
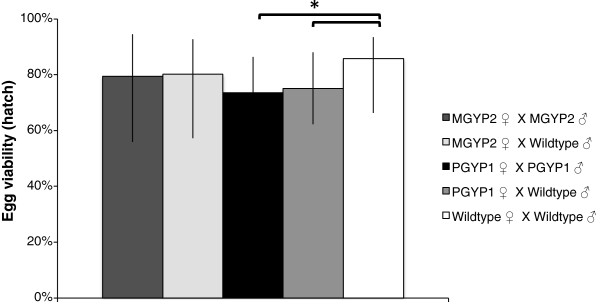
**Egg viability expressed as median hatch rate ± quartiles.** Kolmogorov-Smirnov test is significant if *P < Holm-Bonferroni α (Table 3)*.* The *w*MelPop strain in female *Ae. aegypti* (A.PGYP1.out) reduced the hatch rates of eggs.

Larval nutrition of males (F = 7.551, df = 1, *P* < 0.001), *Wolbachia* infections in females (F = 26.908, df = 4, *P* < 0.001), and interactions between these factors (F = 3.745, df = 4, *P <* 0.05) affected fecundity. Wild-type females laid significantly more eggs than *Wolbachia*-infected females, regardless of male nutrition or infection status. When males were reared on high nutrition, *w*Mel-infected females laid more eggs than *w*MelPop-infected females, irrespective of male infection status. However, when males were reared on low nutrition, no difference was observed between the fecundity of *w*Mel and *w*MelPop-infected females (Figure 1, Table 1). *w*Mel-infected females that mated with males reared on high-nutrition larval diets laid more eggs than those that mated with males reared on low-nutrition larval diets (Table 2). 

**Table 1:** **Summary of statistics comparing the fecundity of *Wolbachia*-infected and uninfected *Ae. aegypti***

**Male larval nutrition**	**Line 1**	**Line 2**	***t***	**df**	***P***	**Holm-Bonferroni *α***
Low	POPO	WTWT	−5.208	149.789	**0.000**	0.005
	POWT	WTWT	−6.447	143.388	**0.000**	0.005
	MOWT	WTWT	−5.731	143.918	**0.000**	0.006
	MOMO	WTWT	−6.069	142.731	**0.000**	0.007
	MOWT	POPO	−0.876	142.470	0.382	0.008
	MOMO	POPO	−0.740	142.995	0.460	0.010
	POPO	POWT	0.610	146.934	0.543	0.012
	MOWT	POWT	−0.414	128.308	0.680	0.017
	MOMO	POWT	−0.212	140.967	0.833	0.025
	MOMO	MOWT	0.209	135.443	0.835	0.050
High	POPO	WTWT	−6.434	197.020	**0.000**	0.005
	POWT	WTWT	−5.771	205.069	**0.000**	0.005
	MOWT	POPO	3.864	182.960	**0.000**	0.006
	MOMO	WTWT	−3.401	184.690	**0.001**	0.007
	MOMO	POPO	3.415	183.289	**0.001**	0.008
	MOWT	WTWT	−3.259	179.891	**0.001**	0.010
	MOWT	POWT	3.077	197.993	**0.002**	0.012
	MOMO	POWT	2.687	195.237	**0.008**	0.017
	MOMO	MOWT	−0.284	166.015	0.777	0.025
	POPO	POWT	−0.773	214.391	0.441	0.050

MGYP2.out females x MGYP2.out males (MOMO), MGYP2.out females x wild-type males (MOWT), A.PGYP1.out females x A.PGYP1.out male (POPO), A.PGYP1.out females x wild-type males (POWT) and wild-type males x wild-type females (WTWT). Unequal variance *t*-test is significant if *P* < Holm-Bonferroni *α*.

**Table 2:** **Summary of statistics comparing the fecundity of *Wolbachia*-infected or uninfected *Ae. aegypti *reared on high or low nutrition diets**

**Male larval nutrition**		***t***	**df**	***P***	**Holm-Bonferroni *α***
**Low**	**High**				
MOMO	MOMO	3.210	137.637	**0.002**	0.010
MOWT	MOWT	−3.364	119.985	**0.001**	0.012
POPO	POPO	0.691	151.755	0.491	0.017
POWT	POWT	−0.681	209.703	0.496	0.025
WTWT	WTWT	0.364	167.047	0.716	0.050

Unequal variance *t*-test is significant if *P* < Holm-Bonferroni *α.*

### Egg viability

**Figure 2:**
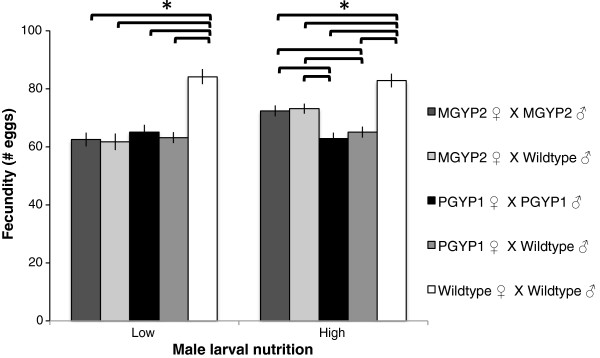
**Mean fecundity ± SEM.** Significant difference between lines *P < Holm-Bonferroni α. Both the *w*Mel (MGYP2.out) and *w*MelPop (A.PGYP1.out) strains of *Wolbachia* reduced the fecundity of infected *Ae. aegypti.*

*w*MelPop infections in females influence the hatch rates of eggs. A generalized linear model (Tweedie with identity link) showed that mating combination (Wald = 17.464, df = 4, *P* < 0.05), but not male nutrition (Wald = 3.081, df = 1, *P* = 0.079) or interactions between these factors (Wald = 3.3173, df = 4, *P* = 0.529), affected the hatch rates of eggs. No significant difference was observed between wild-type females and *w*Mel-infected females regardless of male infection status. More eggs hatched from wild-type females than from *w*MelPop-infected females. No difference was observed between *w*MelPop and *w*Mel-infected females (Figure 2, Table 3).

**Table 3:** **Statistics table comparing hatch rate of eggs of *Wolbachia*-infected and uninfected mosquitoes**

**Line 1**	**Line 2**	***Z***	***P***	**Holm-Bonferroni *α***
POPO	WTWT	2.271	**0.000**	0.005
POWT	WTWT	2.084	**0.000**	0.005
POPO	POWT	1.683	0.007	0.006
MOMO	POPO	1.470	0.027	0.007
MOMO	POWT	1.373	0.046	0.008
MOWT	WTWT	1.231	0.097	0.010
MOMO	WTWT	1.352	0.052	0.012
MOWT	POPO	1.330	0.058	0.017
MOWT	POWT	1.210	0.107	0.025
MOMO	MOWT	0.610	0.850	0.050

Kolmogorov-Smirnov test is significant if *P* < Holm-Bonferroni *α.*

### Re-mating success of males

When males were allowed to mate for a second time, with new cohorts of females, few females were successfully mated ( $\bar{x}$ = 1.6, SEM = 0.280). A generalized linear model (Poisson with log link) showed that *w*MelPop infection (Wald = 0.695, *P* = 0.707), male larval nutrition (Wald = 1.969, *P* = 0.161) and interactions between these factors did not affect the number of females that laid eggs. Generalized linear models (Tweedie with identity link) showed that fecundity (F) and hatch rates of eggs (H) were not significantly affected by *w*MelPop-infection (F: Wald = 1.64, *P* = 0.441; H: Wald = 2.704, *P* = 0.259), male larval nutrition (F: Wald = 0.969, *P* = 0.325; H: Wald = 0.98, *P* = 0.322) or interactions between these factors (F: Wald = 5.089, *P* = 0.078; H: Wald = 0.199, *P* = 0.905). These data suggest that most males depleted themselves in the first mating experiment and did not completely renew mating ability by the second mating.

### Sperm quantity and viability

Male larval nutrition and *Wolbachia* infections do not affect the quantity or viability of sperm. The quantity (Q) and viability (V) of sperm produced by males was not affected by *w*MelPop infection (Q: F = 2.325, *P* = 0.136; V: F = 2.412, *P* = 0.129), male larval nutrition (Q: F = 2.382, *P* =0.131; V: F = 1.79, *P* = 0.189) or interactions between these factors (Q: F = 0.626, *P* = 0.434; V: F = 0.475, *P* = 0.495).

## Discussion

Both *w*MelPop and *w*Mel caused reductions in female fecundity regardless of the infection status of male mates. Males reared on low-nutrition larval diets developed more slowly, were smaller and inseminated fewer females than those reared on high-nutrition larval diets. Interestingly, the fecundity of *w*Mel-infected females was reduced when the mated male, regardless of infection status, had been reared on a low nutrition diet. For *w*MelPop-infected females, fecundity was consistently low, and was not rescued by the rearing of males on high nutrition like in the case of *w*Mel. The reduced fecundity of *w*MelPop is not unexpected given the virulence of the infection. The reduction in fecundity of *w*Mel females due to male nutrition, however, has not been previously reported. *Wolbachia* infection did not affect larval development or male wing length in this study, although, small effects have been reported previously by Yeap *et al*. [50]. Neither strain of *Wolbachia* affected sperm quality or viability or the ability of males to successfully mate a second cohort of females.

Female fecundity measurements taken soon after the establishment of the PGYP1 and MGYP2 lines of *Ae. aegypti* failed to show changes in response to *Wolbachia* infection [2,3]. However, a subsequent investigation examining the effects of diverse human bloods and non-human bloods on the mosquito revealed a fecundity reduction of 27% for PGYP1 [57]. This estimate is consistent with our measure of 23%. The sudden appearance of a fecundity cost in *w*Mel (19% reduction) could possibly be explained by the use of different human blood feeders for characterisation [57]. Alternatively, these temporal changes in the effects of *Wolbachia* infection could reflect an increase in the virulence and or changes in the genetic background of the mosquito line.

The possible mechanistic bases for reductions in fecundity are many. Changes in the blood-feeding behaviour of *Wolbachia*-infected mosquitoes could influence the reduced fecundity of infected females. *Ae. aegypti* seek blood meals to maximise fecundity and reserves [58] and Turley *et al*. [23] showed that *w*MelPop-infected mosquitoes imbibe smaller blood meals than uninfected mosquitoes, limiting the resources available for egg synthesis in *w*MelPop-infected females, and potentially reducing the number and viability of eggs produced. To date, the size of blood meal imbibed by *w*Mel-infected mosquitoes has not been investigated. Therefore, it is possible that the reduced fecundity of *Wolbachia*-infected females in this study could be explained by reduced blood-feeding abilities of *Wolbachia*-infected mosquitoes.

Another potential explanation for the reduced fecundity of *Wolbachia*-infected females is the incorrect processing of male accessory reproductive gland proteins. Nuptial gifts are common in insects and seminal gifts – genitally absorbed male donations – are often beneficial to offspring fitness [59-62]. Male accessory reproductive glands produce secretions essential for the transfer of sperm to females during mating. Proteins in these secretions affect female reproductive activity and improve males’ chances of siring females’ offspring. In many insects, egg production and eventual deposition occur at reduced rates in virgin females compared to mated insects, due to the presence of fecundity-enhancing substances in male accessory reproductive gland secretions [63]. Accessory gland reproductive proteins have been shown to increase egg production in *Aedes* spp. This is thought to be due to stimulation of vitellogenesis, the synthesis and secretion of egg yolk protein precursors by the mosquito fat body [64-67]. Parasites are known to modify vitellogenesis [68,69] and *Wolbachia* infections are associated with decreases in yolk protein gene expression in previtellogeneic ovaries of *Drosophila melanogaster*[70]. Therefore, it is possible that *Wolbachia* infections in *Ae. aegypti* females could interfere with the processing of male accessory reproductive gland proteins, potentially reducing vitellogenesis and host fecundity.

Reduced viability of *w*MelPop eggs could be attributed to increased frequency of apoptosis in the female germline cells. This and previous studies have shown that *w*MelPop infections reduce the hatch rate of eggs laid by *Ae. aegypti*[50,57]. Apoptosis, a form of programmed cell death, is a process needed for normal development and is a feature of female germline development common to vertebrate and invertebrate species [71-74]. A recent study of *D. melanogaster* has shown that *w*MelPop infections increase the frequency of apoptosis in the female germline cells compared to *w*Mel-infected and uninfected lines [75]. Infections with *w*MelPop could induce similar effects in *Ae. aegypti,* potentially impacting the development of embryos such that fewer eggs are viable.

The decreases in reproductive success observed in this study may not be sufficiently strong to prevent the spread of *Wolbachia* into host populations. Population modelling has suggested that up to a 50% reduction in fecundity could be overcome by the expression of strong cytoplasmic incompatibility and still allow spread of *Wolbachia*[76]. In addition, other, less well understood, benefits of *Wolbachia* infection, such as *Wolbachia*-mediated protection against mosquito pathogens, could enhance host fitness. Early studies of *Drosophila simulans* infected with the *Wolbachia* strain *w*Ri showed that females infected with this strain were 10-20% less fecund than *Wolbachia-*cured and wild-type counterparts [77]. Despite this cost, *w*Ri rapidly spread through California populations of *D. simulans* between 1984 and 1994 [6]. Indeed both the field cage trials [3] and the open field releases for *w*Mel [4] have demonstrated effective spread of this strain into *Ae. aegypti* populations. Open field releases of the *w*MelPop only just began in 2012 and are still ongoing.

## Conclusion

This study demonstrates that males infected with *w*Mel or *w*MelPop do not suffer a reduced ability to inseminate females. While males in general suffer fitness consequences when reared on low nutrition that for the most part these effects are not made more extreme by the presence of *Wolbachia*. These findings bode well for the ability of infected males to cause cytoplasmic incompatibility in populations of mixed infection status as in open field releases. Surprisingly, *w*Mel infected females appear to suffer similar reductions in fecundity as *w*MelPop-infected females, an effect exacerbated by rearing of male mates in low nutrition environments. These reductions in fitness, however, are within the range of what may be mitigated by the expression of cytoplasmic incompatibility still allowing for *Wolbachia* infections to spread.

## Competing interests

The authors declare they have no competing interests.

## Authors’ contribution

AT, MZ, SO and EM designed the study. AT carried out the research. AT, MZ and EM analyzed the data. AT drafted the manuscript. All authors read and approved the final manuscript.
